# Ginsenoside Rh2- functionalized liposomes enhanced BRD4-PROTAC delivery and antitumor efficacy *via* improved tumor targeting and ECM remodeling

**DOI:** 10.1016/j.mtbio.2026.102767

**Published:** 2026-01-05

**Authors:** Lijuan Wen, Jialei Rao, Jiaoting Chen, Fang Li, Xixi Chen, Shenpeng Guo, Binghui Cui, Caisheng Qiu, Weiliang Chen

**Affiliations:** a Key Laboratory of Prevention and Treatment of Cardiovascular and Cerebrovascular Diseases of Ministry of Education, Gannan Medical University, University Park in Rongjiang New District, Ganzhou, 341000, People's Republic of China; b College of Pharmacy, Gannan Medical University, University Park in Rongjiang New District, Ganzhou, 341000, People's Republic of China; c Department of Pharmacy, Children's Hospital of Soochow University, Suzhou, 215003, People's Republic of China

**Keywords:** PROTAC, BRD4, Ginsenoside Rh2, Liposomes, ECM remodeling

## Abstract

PROTAC technology leverages the ubiquitin-proteasome system to selectively degrade target proteins, presenting a novel strategy for anticancer therapy. ARV825, a BRD4-targeting PROTAC, exerts potent antitumor effects by degrading BRD4, thereby suppressing Bcl-2 and PD-L1 expression, inducing apoptosis, and enhancing T cell-mediated immunity. However, its clinical translation is hindered by poor solubility, low membrane permeability, and off-target effects. While conventional liposomes (lip) improved ARV825 delivery, their efficacy remained limited by insufficient tumor targeting and collagen-rich extracellular matrix (ECM) barriers that restricted T cell infiltration. To address these challenges, ginsenoside Rh2 (GRh2)-functioned liposomes (Gip) were developed by replacing cholesterol with GRh2. Gip exhibited high drug encapsulation efficiency and superior stability. *In vitro*, Gip significantly improved cellular uptake in 4T1 cells and 3D tumor spheroids *via* GLUT1-mediated transport, leading to more efficient BRD4 degradation and greater cytotoxicity than lip. *In vivo*, Gip demonstrated superior tumor accumulation in subcutaneous and lung metastasis models, owing to its active targeting capability. Crucially, GRh2-mediated collagen degradation synergized with ARV825-induced PD-L1 suppression to enhance T cell infiltration. As a result, ARV@Gip exhibited superior antitumor efficacy through dual mechanisms, including enhanced apoptosis and immune activation, outperforming ARV@lip in both tumor models. Collectively, this GRh2-functionalized liposomal platform overcomes key pharmacological barriers by integrating enhanced tumor targeting, ECM modulation, and dual pro-apoptotic/immunostimulatory effects, offering a promising therapeutic strategy for breast cancer.

## Graphical abstract

**Figure:**
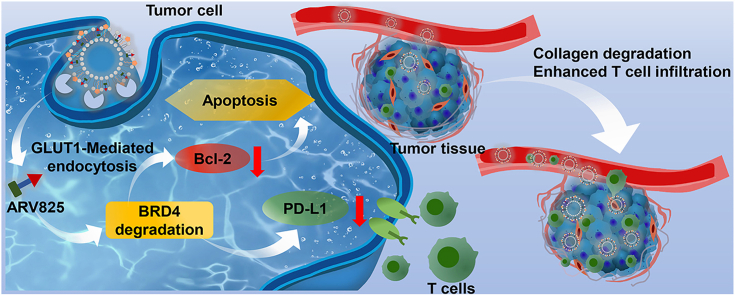

## 1 Introduction

Cellular proliferation and metastatic potential in tumors are critically dependent on the precise regulation of protein functions. Targeted protein degradation has emerged as a promising therapeutic strategy, wherein proteolysis-targeting chimeras (PROTACs) representing a novel class of bifunctional molecules that exploit the ubiquitin-proteasome system (UPS) for selective protein elimination [1,2]. PROTACs consist of three key components: a target protein-binding ligand, an E3 ubiquitin ligase-recruiting moiety, and a connecting linker. This unique architecture enables PROTACs to facilitate polyubiquitination and subsequent proteasomal degradation of target proteins, distinguishing them from traditional small-molecule inhibitors that merely block protein activity through occupancy-driven mechanisms [[3], [4], [5]].

The event-driven catalytic nature of PROTACs offers several pharmacological advantages, including sustained target suppression at sub-stoichiometric concentrations and reduced susceptibility to drug resistance. Despite these theoretical benefits, clinical translation of PROTAC technology has been slow, with only a handful of candidates progressing to phase II clinical trials after more than a decade of research. This limited progress primarily stems from inherent physicochemical challenges of PROTACs, including poor aqueous solubility, inadequate membrane permeability, potential off-target effects, and the paradoxical HOOK effect observed at higher concentrations [[6], [7], [8]].

Nanoparticle-based delivery systems have emerged as a viable strategy to overcome the limitations of PROTACs mentioned above [[9], [10], [11]]. Among various nanocarriers, liposomes-self-assembled phospholipid vesicles structurally analogous to biological membranes - have shown particular promise [12,13]. Their clinical validation is evidenced by FDA-approved formulations such as doxorubicin liposomes. The amphiphilic features of liposomes enabled efficient encapsulation of hydrophobic compounds within their lipid bilayers, while their nanoscale dimensions facilitate tumor accumulation through the enhanced permeability and retention (EPR) effect [14]. In our previous work, we demonstrated that liposomal encapsulation could significantly improve the solubility and tumor-targeting efficiency of ARV825, a BRD4-targeting PROTAC [15].

BRD4, a member of the bromodomain and extraterminal (BET) protein family, plays a pivotal role in oncogenesis by regulating the expression of key survival factors including Bcl-2 and immune checkpoint protein PD-L1 [[16], [17], [18], [19], [20]]. Our prior studies in triple-negative breast cancer models revealed that while conventional liposomal ARV825 delivery could induce BRD4 degradation and subsequent downregulation of Bcl-2 and PD-L1, the therapeutic efficacy remained suboptimal. This limitation was attributed to two major factors: (1) insufficient tumor-specific accumulation relying solely on passive EPR-mediated targeting, and (2) the dense collagen matrix in tumor extracellular matrix (ECM) that physically impedes T-cell infiltration [15].

To address these challenges, we developed a novel liposomal system functioned with ginsenoside Rh2 (GRh2), a bioactive saponin derived from *Panax ginseng* with established immunomodulatory properties (marketed in China as Jinxing capsules). GRh2's structural similarity to cholesterol enables its integration into liposomal membranes, enhancing formulation stability. More importantly, the glucose moiety of GRh2 facilitates active tumor targeting through glucose transporter-1 (GLUT1) recognition, which is frequently overexpressed in breast cancer cells [21,22]. Beyond its targeting function, GRh2 exhibits multifaceted antitumor activity, including induction of apoptosis, degradation of tumor collagen, and promotion of immune cell infiltration [23,24]. Preliminary studies from Wang's group demonstrated that GRh2-functionalized liposomes significantly improved the tumor-targeting efficiency and therapeutic index of paclitaxel compared to conventional formulations [25,26].

**Scheme 1:**
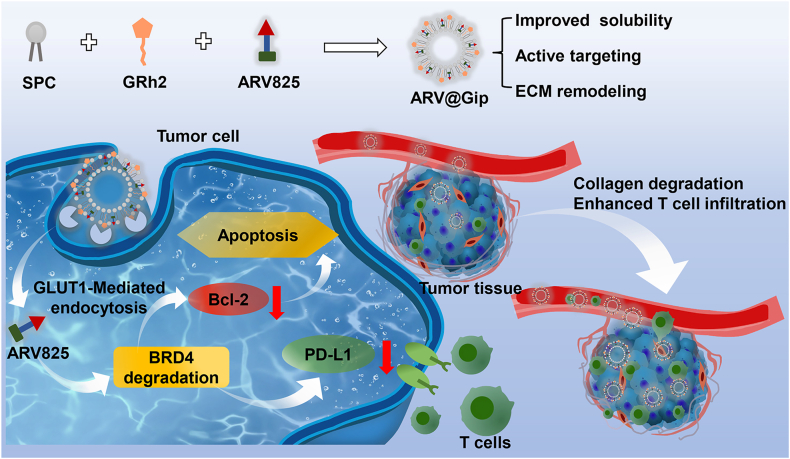
GRh2-functionalized liposomes enhanced BRD4-PROTAC delivery and antitumor efficacy *via* improved tumor targeting and ECM remodeling.

In the current study, we hypothesize that GRh2-modified liposomes will overcome the delivery challenges of BRD4-PROTACs through a combination of passive and active targeting mechanisms (Scheme 1). The proposed system is designed to: (1) enhance PROTAC solubility and membrane permeability, (2) improve tumor accumulation *via* EPR effect and GLUT1-mediated active targeting, (3) promote T-cell infiltration through GRh2-mediated collagen degradation, and (4) simultaneously induce tumor cell apoptosis and restore antitumor immunity *via* BRD4 degradation. We anticipate that the active targeting capability of GRh2-functionalized liposomes, combined with the intrinsic collagen-degrading activity of GRh2 and the PD-L1 downregulating effect of ARV825, will work in concert. Consequently, this multi-mechanism approach is expected to achieve a synergistic antitumor efficacy. Through comprehensive evaluation of formulation characteristics, targeting efficiency, and therapeutic efficacy in both subcutaneous tumor models and lung metastases models, we aim to establish a clinically translatable platform for PROTAC delivery in breast cancer treatment.

## 2 Materials and method

### 2.1 Materials

Soy phosphatidylcholine (SPC), Cholesterol, 1,1′-Dioctadecyl-3,3,3′,3′-Tetramethylindotricarbocyanine Iodide (DiR), and 3-(4,5-Dimethylthiazol-2-yl)-2,5- diphenyltetrazolium bromide (MTT) were purchased from Maklin Reagent, China. GRh2 and ARV825 was purchased from Bidepharm, China. Primary antibodies (BRD4, Bcl-2 and PD-L1) were sourced from Abcam, USA. The CD8^+^ antibody labeled with FITC was acquired from Thermo Fisher Scientific. The live/dead staining kit, ATP assay kit, and TdT-mediated dUTP nick end labeling (TUNEL) staining kit were obtained from Beyotime Biotechnology. The Annexin V-FITC cell apoptosis kit was purchased from Solarbio Science & Technology.

4T1 cells labeled with Luc (4T1-Luc) was purchased from Procell Biotech and cultured with 1640 medium (Containing 10 % FBS and 1 % penicillin streptomycin) at 37 °C with 5 % CO_2_.

Female Balb/c mice, aged 4–6 weeks, were obtained from Gempharmatech Co., Ltd and housed in the SPF Animal Laboratory in Gannan Medical University.

### 2.2 Preparation of ARV@Gip

The ARV825-loaded GRh2-functionalized liposomes (ARV@Gip) were prepared using an optimized thin-film hydration technique. A precisely measured lipid mixture containing soy phosphatidylcholine (SPC, 50 mg), GRh2 (10 mg), and ARV825 (1 mg) was completely dissolved in 5 mL of a dichloromethane: methanol (1:1, v/v) cosolvent system. The organic phase was subsequently removed by rotary evaporation to generate a uniform lipid film, which was then hydrated with distilled water and subjected to probe sonication (20 % output power, 5 min total duration with 2 s on/off pulse cycles) followed by sequential extrusion through a 0.22 μm polyethersulfone membrane to yield the final liposomes [26].

For comparative studies, three control formulations were prepared following identical protocols: (1) blank GRh2-functionalized liposomes (Gip) without ARV825 loading, (2) conventional ARV825-loaded liposomes (ARV@lip) with cholesterol (10 mg) substituted for GRh2, and (3) blank conventional liposomes (lip) containing cholesterol (10 mg) instead of GRh2.

### 2.3 Characterization of ARV@Gip

The hydrodynamic diameter and zeta potential of the liposomal formulations were analyzed using dynamic light scattering (DLS) with a Zetasizer Nano S90 (Malvern Instruments, UK).

Encapsulation efficiency (EE%) and drug loading capacity (DL%) were quantified *via* an ultrafiltration-centrifugation method (MWCO = 3 KDa) and free ARV825 was separated from encapsulated drug by centrifugation. The concentration of ARV825 was determined by high-performance liquid chromatography (HPLC) [27]. The EE% and DL% were calculated by the following equation.

$$\text{EE}\%=\left(W_{\text{total}\;\text{drug}}-W_{\text{free}\;\text{drug}}\right)/\;W_{\text{total}\;\text{drug}}\times 100\%$$

$$\text{DL}\%=\left(W_{\text{total}\;\text{drug}}-W_{\text{free}\;\text{drug}}\right)/\;\left(W_{\text{total}\;\text{drug}}+W_{\text{total}\;\text{lipd}}\right)\times 100\%$$

where W_total drug_, W_free drug_ and W_total lipd_ referred to the weight of free drug, total drug and lipids respectively [28].

### 2.4 Stability and release study of ARV@Gip

The colloidal stability and drug retention of ARV@Gip and ARV@lip were evaluated under refrigerated storage conditions (4 °C). Hydrodynamic diameter, polydispersity index (PDI), zeta potential and encapsulation efficiency (EE%) were analyzed at predetermined intervals (days 1, 2, 3, 5, and 7) using dynamic light scattering (DLS, Zetasizer Nano S90) and the ultrafiltration-HPLC method described in Section 2.3 [21]. Additionally, the stability of ARV@Gip and ARV@lip in PBS (at 4 °C) and in 10 % FBS (at 37 °C) was also investigated by measuring their particle size, PDI, and zeta potential.

The *in vitro* drug release profiles of ARV@lip and ARV@Gip were investigated using a dialysis method. Liposome dispersions were placed in dialysis bags (MWCO = 3 kDa) and immersed in release medium (PBS, pH 7.4) under gentle agitation at 37 °C. At predetermined time intervals, samples were withdrawn from the release medium, and the concentration of released ARV825 was quantified by HPLC.

### 2.5 Cellular uptake Investigation

To track cellular internalization, coumarin-6 (C6), a lipophilic fluorescent probe, was encapsulated into both Gip and lip using the film-hydration methods [29]. For quantitative analysis, 4T1 cells were seeded in 6-well plates at a density of 1 × 10^6^ cells/well and subsequently incubated with either C6-loaded lip (C6@lip) or C6-loaded Gip (C6@Gip) for 6 h. Following incubation, cells were harvested and subjected to quantitative analysis using flow cytometry (FACS Calibur, BD Biosciences).

For spatial localization assessment, parallel experiments were conducted for confocal microscopic observation. Post-incubation with the nanoparticle formulations, cells were fixed with 4 % paraformaldehyde (PFA) and nuclear counterstaining was performed using 4′,6-diamidino-2-phenylindole (DAPI). Cellular internalization patterns were visualized using confocal laser scanning microscopy (CLSM, ZEISS 880).

### 2.6 BRD4 protein degradation, Bcl-2 and PD-L1 protein down-regulation

Target protein expression levels (BRD4, Bcl-2, and PD-L1) were quantitatively evaluated through western blot analysis to assess the molecular mechanisms underlying therapeutic efficacy [30]. In brief, 4T1 cells were seeded in 6 well plate at a density of 10^6^ cells/well and incubated with PBS, lip, Gip, ARV@lip or ARV@Gip (concentration of ARV825 = 1 μg/mL) for 48 h. Later, the total proteins in tumor cells were collected and analyzed using western blot.

### 2.7 Cytotoxicity assessment

The cytotoxic effects of ARV@Gip on 4T1 cells were evaluated using the MTT assay [30]. Briefly, cells were seeded in 96-well plates at a density of 1 × 10^3^ cells/well and allowed to adhere for 24 h. Subsequently, cells were treated with varying concentrations of ARV@Gip for 48 h. Following treatment, 20 μL of MTT solution (5 mg/mL) was added to each well and incubated for 4 h. The resulting formazan crystals were dissolved in 150 μL DMSO, and absorbance was measured at 490 nm using a microplate reader. Cell viability was calculated as follows:

$$\text{Cell}\;\text{viability}\;\left(\%\right)=\left(A_{\text{treated}}-A_{\text{blank}}\right)\;/\;\left(A_{\text{Non}-\text{treated}}-A_{\text{blank}}\right)\times 100$$

where A_treated_, A_non-treated_, and A_blank_ represent the absorbance values of drug-treated, untreated (control), and blank wells, respectively. The cytotoxic effects of other groups (ARV825, ARV@lip, lip and Gip) against 4T1 cells was also studied.

Besides, the cytotoxic effects of various formulations, including ARV825-loaded liposomes (ARV@lip and ARV@Gip) and their corresponding blank carriers (lip and Gip), were evaluated on 4T1 cells, normal mouse hepatocytes (AML12), and mouse fibroblast cells (L929) using the MTT assay.

### 2.8 Live/dead cell viability assay

Cell viability following ARV@Gip treatment was assessed using a calcein-AM/propidium iodide (PI) viability/cytotoxicity kit [31]. Briefly, 4T1 cells were seeded in 24-well plates and treated with PBS, lip, Gip, ARV@lip, or ARV@Gip (ARV825 concentration: 1 μg/mL) for 48 h. Cells were then stained with calcein-AMand PI for 30 min. Fluorescence microscopy was performed to visualize viable (green, calcein-AM-positive) and dead (red, PI-positive) cell populations.

### 2.9 Evaluation of liposome permeability in tumor spheroids

Three-dimensional (3D) tumor spheroids were generated by seeding 4T1 cells (1 × 10^3^ cells/well) in 96-well ultra-low attachment plates and culturing for 5 days [32]. To assess liposome penetration, mature spheroids were incubated with coumarin-6-labeled formulations (C6@lip or C6@Gip; C6 concentration: 0.2 μg/mL) for 6 h. Fluorescent probe distribution across spheroid depth was analyzed *via* CLSM (step size: 20 μm).

### 2.10 Therapeutic efficacy evaluation of ARV@Gip in 3D tumor spheroids

3D tumor spheroids were generated as previously described (Section 2.9) and subjected to treatment with either blank medium, lip, Gip, ARV@lip, or ARV@Gip (ARV825 concentration: 1 μg/mL) upon reaching a diameter of 300 μm. Spheroid morphology was daily monitored by microscopy [33].For quantitative viability assessment, spheroids were incubated with 10 μL CCK-8 reagent (5 mg/mL) per well for 4 h at 37 °C on day 6.

### 2.11 *In vivo* biodistribution analysis

The tumor-targeting capability of Gip was evaluated using near-infrared (NIR) imaging with DiR used as an NIR dye [34]. Subcutaneous 4T1 tumor models were established in Balb/C mice by inoculating 1 × 10^6^ cells into the right flank. When tumors reached ∼100 mm^3^, mice received intravenous injections of DiR-labeled ARV@lip (DiR@lip) or ARV@Gip (DiR@Gip) *via* the tail vein. Whole-body fluorescence imaging (IVIS Spectrum, PerkinElmer) was performed at predetermined intervals. At 48 h post-injection, mice were euthanized, and major organs (heart, liver, spleen, lung, kidney) and tumors were excised for *ex vivo* imaging to quantify nanoparticle accumulation.

### 2.12 *In vivo* protein modulation analysis

BRD4 degradation and Bcl-2/PD-L1 downregulation in tumor tissues were assessed *via* immunofluorescence staining. Tumor-bearing mice (tumor volume: ∼100 mm^3^) received PBS, ARV@lip, or ARV@Gip *via* tail vein injection. At 24 h post-treatment, tumors were harvested, snap-frozen, and sectioned into 10 μm cryosections. After fixation and permeabilization, sections were incubated with primary antibodies against BRD4, Bcl-2 and PD-L1, followed by incubation with Alexa Fluor 488 or Cy5-conjugated secondary antibodies. Nuclei were counterstained with DAPI, and the sections were observed using CLSM.

### 2.13 *In vivo* antitumor efficacy of ARV@Gip in subcutaneous tumor models

Subcutaneous 4T1-Luc tumor models were established in female Balb/C mice by injecting 1 × 10^6^ cells into the right axillary region. When tumor volumes reached approximately 100 mm^3^, the tumor-bearing mice were randomly allocated into five treatment groups receiving intravenous injections *via* the tail vein on days 1, 4, 7, and 10: (1) PBS, (2) lip, (3) Gip, (4) ARV@lip, or (5) ARV@Gip (Dose of ARV825 was 8 mg/kg). Tumor dimensions and body weights were monitored every 3 days, with tumor volume calculated using the formula: V = (length × width^2^)/2. On day 16 post-treatment initiation, animals were euthanized for terminal analysis. Excised tumors were imaged, weighed, and processed for histological evaluation.

Tumor tissues underwent hematoxylin and eosin (H&E) staining to assess morphological changes and necrosis extent. The distribution of collagen in tumor tissues was evaluated by Masson's trichrome staining. Apoptotic cells were studied using TUNEL staining, while tumor-infiltrating CD8^+^ T lymphocytes were evaluated by immunofluorescence staining. To comprehensively quantify the tumor immune microenvironment, we performed flow cytometry analysis on tumor-infiltrating immune cells, with a specific focus on CD4^+^ T cells, CD8^+^ T cells, and regulatory T cells (Tregs). . Serum biochemical parameters (ALT, AST, BUN, CREA) were measured, and histopathological examination (H&E staining) of major organs (heart, liver, spleen, lungs, and kidneys) was performed in mice after the completion of the treatment regimen.

### 2.14 Tumor targeting of Gip in lung metastasis

Lung metastasis models of breast cancer were established by intravenous injection of 1 × 10^6^ 4T1-Luc cells into female Balb/C mice *via* the tail vein [35]. Tumor engraftment was confirmed by bioluminescence imaging following intraperitoneal administration of D-luciferin potassium salt (120 mg/kg) using an IVIS imaging system. To evaluate tumor-targeting efficiency, mice were intravenously injected with either DiR@lip or DiR@Gip. NIR fluorescence imaging was performed 48 h post-injection to assess biodistribution. Subsequently, mice were euthanized, and major organs (heart, liver, spleen, lungs, and kidneys) were excised for *ex vivo* bioluminescence and NIR fluorescence imaging to quantify liposome accumulation.

### 2.15 Evaluation of ARV@Gip antitumor efficacy in lung metastasis models

Bioluminescent 4T1-Luc lung metastasis models were established and the tumor-bearing mice were randomly allocated into four experimental groups receiving tail vein administrations on days 1, 4, and 7: (1) PBS, (2) Gip, (3) ARV@lip and (4) ARV@Gip. All drug-treated groups received an equivalent dose of 8 mg ARV825 per kg body weight. Tumor progression was monitored using *in vivo* bioluminescence imaging on day 1, 4, 7 and 10 [35,36]. Body weight was measured as a surrogate for systemic toxicity on days 1, 4, 7 and 10 using a precision electronic balance. On day 10, mice were humanely euthanized, and lungs were excised for *ex vivo* bioluminescence imaging to quantify metastatic burden. And H&E staining of lung tissues was carried out to study the distribution of tumor cells.

### 2.16 Statistical analysis

All the data were presented as Mean ± SD. Significant difference analysis used a one-way ANOVA or a paired T test, described as follows: ∗ (*p* <0.05), ∗∗ (*p* <0.01) and ∗∗∗ (*p* <0.001).

## 3 Results and discussion

### 3.1 Preparation, characterization and stability of ARV@Gip

The liposomal formulation was prepared using the film-hydration method with GRh2 as a cholesterol substitute. Structurally analogous to cholesterol, GRh2 effectively integrates into SPC bilayers through its hydrophobic moiety, mirroring cholesterol's membrane-stabilizing properties. Building upon previous reports demonstrating the successful application of Gip for hydrophobic drug (such as paclitaxel) delivery [21,26], we encapsulated the PROTAC-based antitumor agent ARV825 within this system to generate ARV825-loaded Gip (ARV@Gip).

**Fig. 1:**
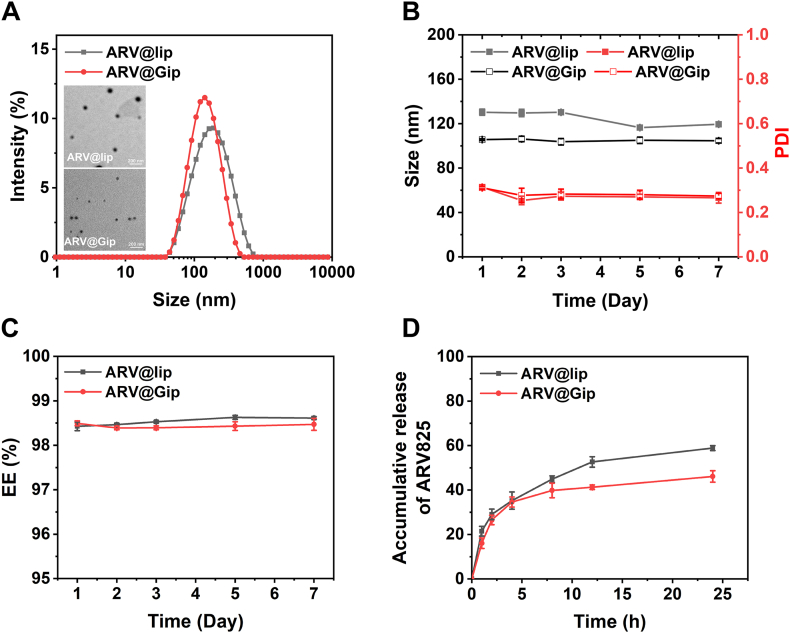
Physicochemical characterization of ARV@Gip. (A) Size distribution profiles and TEM images of ARV@Gip compared with conventional ARV@lip. (B) Temporal stability assessment: Particle size and PDI of ARV@Gip and ARV@lip over 7 days of storage in water at 4 °C. (C) EE% stability of ARV@Gip during 7-day storage in water at 4 °C. (D) *In vitro* drug release kinetics of ARV@Gip and ARV@lip in PBS (pH 7.4).

Physicochemical characterization revealed that ARV@Gip shared comparable properties with conventional ARV@lip, including high EE%, substantial DL%, and a negative zeta potential (Table 1). Notably, ARV@Gip exhibited a marginally smaller particle size (∼100 nm) than ARV@lip (Fig. 1A), consistent with prior reports on GRh2-modified liposomes [21]. The incorporation of ARV825 had negligible impact on the size distribution or surface charge of the blank Gip liposomes, suggesting stable drug incorporation within the phospholipid bilayer.

**Table 1:** Characterization of ARV@Gip

Formulation	Particle size (nm)	PDI	Zeta potential (mV)	EE (%)	DL (%)
ARV@lip	130.3 ± 2.8	0.31 ± 0.01	−42.5 ± 0.8	98.4 ± 0.1	1.97 ± 0.02
ARV@Gip	107.9 ± 2.4	0.31 ± 0.01	−46.9 ± 0.9	98.5 ± 0.1	1.98 ± 0.02
lip	145.2 ± 0.8	0.29 ± 0.01	−49.7 ± 0.1	/	/
Gip	105.8 ± 3.0	0.29 ± 0.01	−55.8 ± 1.2	/	/

Stability assessments conducted at 4 °C over a 7-day period in water demonstrated excellent preservation of particle size, PDI, EE%, and zeta potential (Fig. 1B–C, Fig. S1). Notably, while the particle size of ARV@Gip was slightly smaller in PBS and 10 % FBS (prepared in PBS) compared to that in water, the formulation still exhibited excellent stability in these physiologically relevant media. No significant changes in particle size, PDI, or zeta potential were observed in PBS at 4 °C over 7 days or in 10 % FBS at 37 °C over 3 days (Fig. S1). These results collectively confirm that GRh2 provides bilayer stabilization comparable to cholesterol. This observation aligns with established literature documenting GRh2's capacity to enhance membrane rigidity and promote drug retention in phospholipid vesicles [21,22].

*In vitro* release studies performed under physiologically relevant conditions (PBS, pH 7.4) revealed sustained release kinetics for ARV@Gip, with less than 45 % of the payload released within 24 h (Fig. 1D). This controlled release profile, similar to that observed for ARV@lip, was particularly advantageous for minimizing premature drug leakage during systemic circulation while facilitating tumor accumulation through the EPR effect. The maintained release kinetics not only ensure adequate drug retention during transit but also serve to mitigate potential off-target effects on healthy tissues [37].

### 3.2 Cellular uptake and BRD4 degradation effect of ARV@Gip

**Fig. 2:**
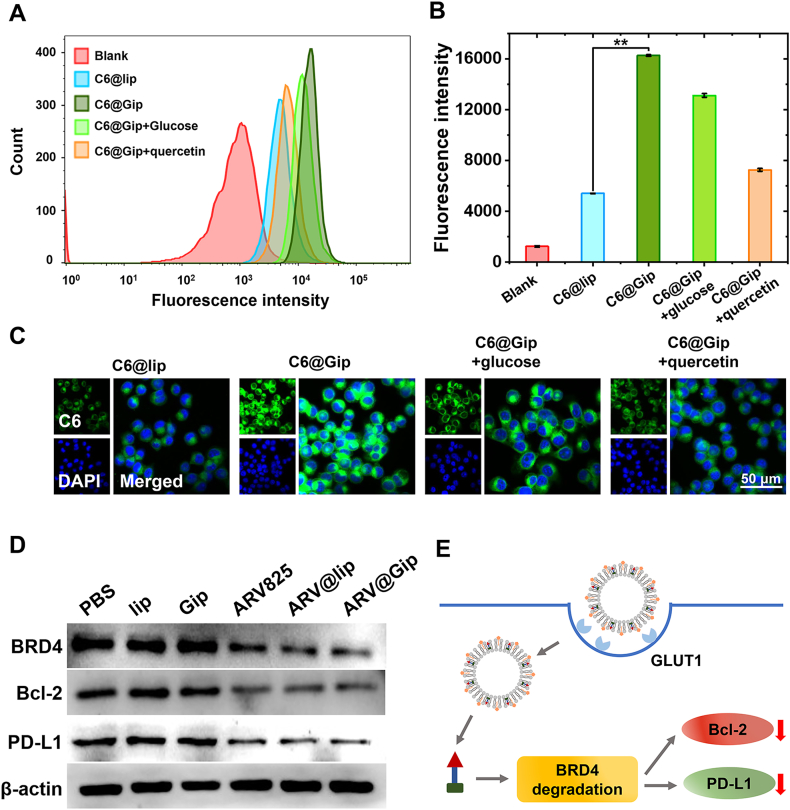
Cellular internalization and BRD4 degradation efficacy mediated by ARV@Gip. (A) Quantitative analysis of cellular uptake for C6-labeled formulations *via* flow cytometry. (B) Comparative fluorescence intensity in 4T1 cells treated with C6-labeled liposomes. (C) CLSM visualization of cellular uptake. (D) Western blot analysis of BRD4, Bcl-2, and PD-L1 protein expression in treated 4T1 cells. (E) Schematic mechanism: Gip-mediated enhancement of cellular internalization and PROTAC efficacy against BRD4.

Cellular uptake efficiency was quantitatively analyzed using flow cytometry with C6 as a fluorescent tracer. As demonstrated in Fig. 2A and B, C6@Gip showed significantly enhanced cellular internalization compared to C6@lip, with higher mean fluorescence intensity in treated cells. CLSM further confirmed these findings (Fig. 2C), revealing substantially stronger cytoplasmic fluorescence in 4T1 cells treated with C6@Gip compared to C6@lip. This mechanism was further confirmed by a significant reduction in C6@Gip uptake upon pretreatment with GLUT1 competitive inhibitors (glucose or quercetin), suggesting GLUT1-mediated endocytosis as the primary internalization pathway. In addition, molecular docking studies provided structural insights, revealing that GRh2, with its glycosyl group, possesses a superior binding affinity for GLUT1 compared to cholesterol (Fig. S2). This enhanced cellular permeability through glucose transporter-mediated uptake may facilitate improved intracellular delivery of ARV825 [38]. This enhanced cellular permeability through glucose transporter-mediated uptake may facilitate improved intracellular delivery of ARV825.

Western blot analysis was performed to evaluate the therapeutic effects on target proteins (Fig. 2D). Neither blank lip nor Gip formulations significantly affected BRD4, Bcl-2, or PD-L1 expression levels. However, ARV825 formulations (ARV825, ARV@lip and ARV@Gip) induced BRD4 degradation, subsequently downregulating downstream proteins Bcl-2 and PD-L1. Importantly, ARV@Gip demonstrated superior efficacy compared to ARV@lip and ARV825, likely attributable to its enhanced cellular uptake. The observed downregulation of anti-apoptotic Bcl-2 and immune checkpoint PD-L1 suggested dual therapeutic benefits: promotion of tumor cell apoptosis and enhancement of antitumor immunity [39,40]. These results indicate that Gip-mediated delivery could potentiate the therapeutic effects of ARV825 through improved cellular internalization and subsequent PROTAC activity (Fig. 2E).

### 3.3 Cytotoxicity and apoptosis effect of ARV@Gip against 4T1 cells

**Fig. 3:**
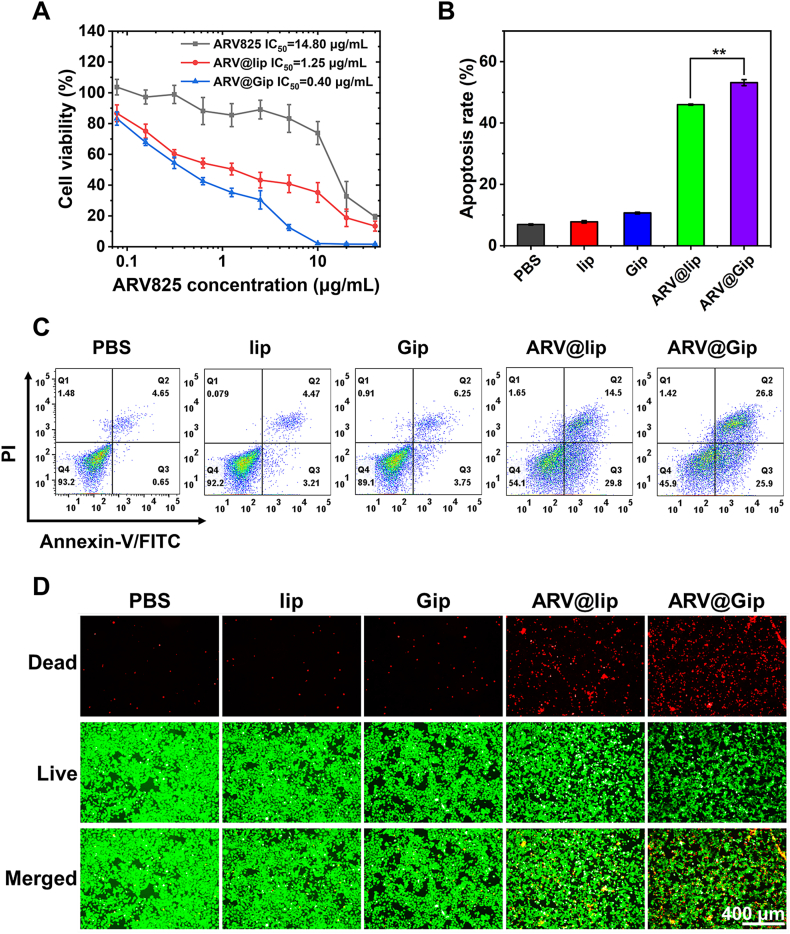
Cytotoxic and pro-apoptotic effects of ARV@Gip in 4T1 cells. (A) Dose-dependent cytotoxicity evaluated by MTT assay. (B) Apoptotic cell quantification *via* Annexin V-FITC/PI staining. (C) Flow cytometric apoptosis profiles. (D) Live/Dead assay (calcein-AM/PI staining) demonstrating treatment-induced cell death.

To evaluate whether ginsenoside-functionalized liposomes enhance the antitumor activity of ARV825, we first assessed cytotoxicity using the MTT assay. As shown in Fig. S3, blank lip exhibited negligible cytotoxicity against 4T1 cells, while Gip showed obvious cytotoxicity at higher concentrations (>50 μg/mL). Both ARV825-loaded formulations (ARV@lip and ARV@Gip) demonstrated concentration-dependent cytotoxicity, with ARV@Gip displaying significantly enhanced potency (Fig. 3A). Notably, ARV@Gip achieved a lower IC_50_ value (0.40 μg/mL) compared to ARV@lip (IC_50_ = 1.25 μg/mL), confirming superior therapeutic efficacy.

These results were further validated by apoptosis assays and live/dead cell viability staining (Fig. 3B–D). Flow cytometry revealed a significantly higher apoptosis rate in ARV@Gip-treated cells than in the ARV@lip group. Consistently, live/dead staining showed a greater proportion of non-viable cells in the ARV@Gip-treated group, aligning with its enhanced cytotoxic profile.

Having determined the IC_50_ values of ARV825 and Gip (Fig. S3) (14.80 and 46.54 μg/mL, respectively), we calculated their combination index (CI) according to the Chou-Talalay method. The resulting CI value of 0.0102, which is far less than 1, indicates a strong synergistic effect between the two agents. This synergism can be attributed to the markedly improved cellular internalization and intracellular delivery of ARV825 mediated by the Gip carrier. The consequent increase in intracellular drug accumulation promoted more efficient BRD4 degradation, leading to the downregulation of the anti-apoptotic protein Bcl-2 and a subsequent amplification of tumor cell death [41]. This mechanistic synergy underscores the potential of Gip as a superior nanocarrier for PROTAC-based antitumor therapy.

Although neither ARV@lip nor ARV@Gip exhibited significant toxicity toward normal hepatocytes (AML12) at concentrations up to 40 μg/mL(Fig. S4), they demonstrated distinct effects on fibroblasts (L929). Specifically, L929 cells showed significantly higher cellular uptake of C6@Gip compared to C6@lip. Consequently, both blank Gip and ARV@Gip potently inhibited the viability of L929 cells (Fig. S5). Based on these findings, we hypothesize that Gip and ARV@Gip may suppress the secretion of collagen fibers within the tumor stroma by targeting and inhibiting the growth of fibroblasts.

### 3.4 Penetration capability and antitumor efficacy of ARV@Gip in 3D tumor spheroids

**Fig. 4:**
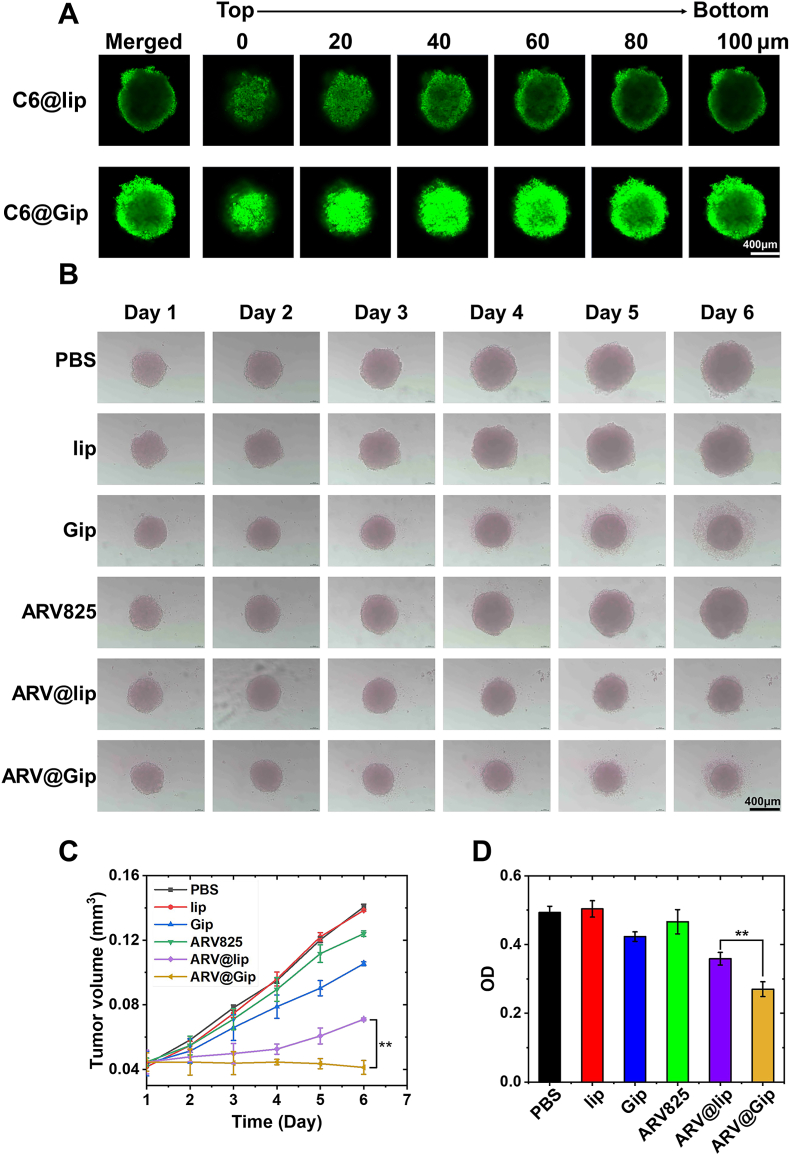
Tumor penetration and growth inhibition in 3D spheroid models. (A) Depth penetration analysis of C6-labeled formulations in tumor spheroids (CLSM z-stacking). (B) Morphological changes in spheroids following treatment. (C) Spheroid volume quantification over time. (D) Growth inhibition rates determined by CCK-8 assay.

The penetration capacity and antitumor effects of GRh2-functionalized liposomes were further evaluated using 3D tumor spheroids. As shown in Fig. 4A, both C6@lip and C6@Gip demonstrated spheroid penetration, as evidenced by detectable green fluorescence throughout the structures. However, C6@Gip exhibited significantly stronger fluorescence intensity in the spheroid core compared to C6@lip, suggesting enhanced penetration efficiency likely attributable to improved cellular uptake mechanisms (as demonstrated in Section 3.2) [42].

Spheroid growth inhibition was monitored to assess therapeutic efficacy. Blank lip showed negligible effects on spheroid volume, while Gip alone induced modest growth suppression. Notably, ARV@Gip treatment resulted in significantly smaller spheroid volumes compared to ARV@lip and free ARV825 (Fig. 4B and C), consistent with its superior cytotoxicity observed in monolayer cultures. On day 6, CCK-8 assays revealed the lowest viability in ARV@Gip-treated spheroids (Fig. 4D), confirming its enhanced potency. These results collectively demonstrate that GRh2 functionalization enhanced both tumor penetration and intracellular drug delivery, leading to amplified BRD4 degradation and Bcl-2 down-regulation (as shown in Section 3.2) and consequent suppression of spheroid growth. The improved penetration-depth-to-efficacy correlation underscored the therapeutic advantage of Gip-based nanocarriers in targeting solid tumor architectures.

### 3.5 Tumor target on subcutaneous tumors and BRD4 degradation effect of ARV@Gip

**Fig. 5:**
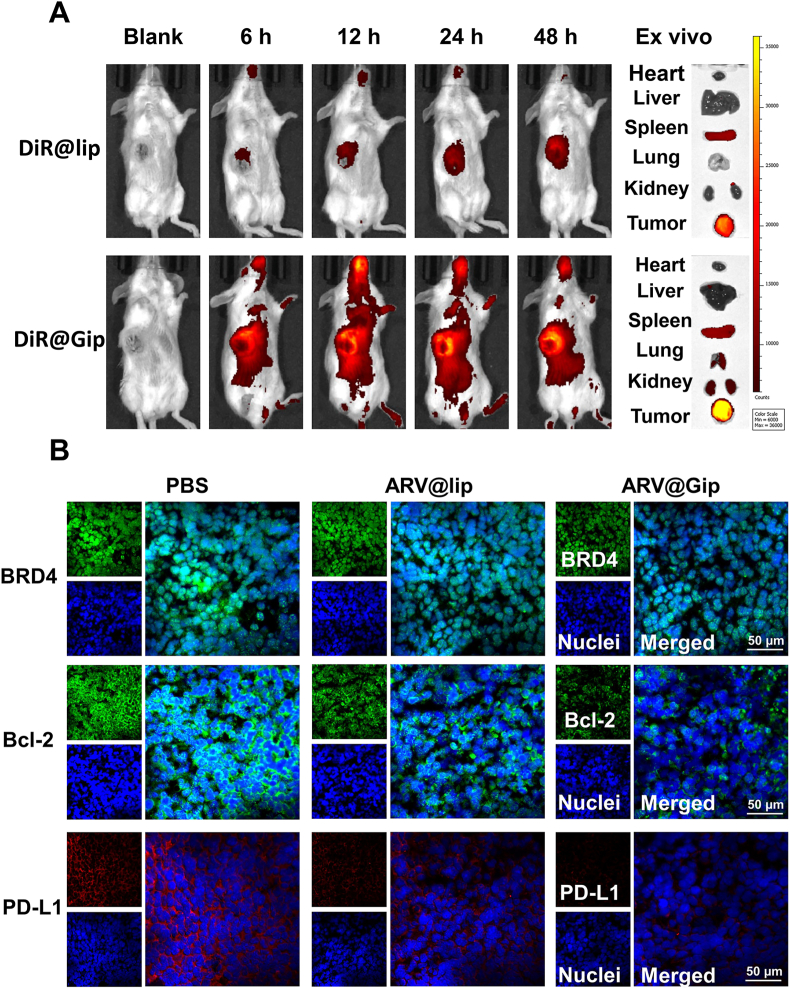
*In vivo* tumor targeting and BRD4 degradation. (A) Biodistribution of DiR-labeled liposomes in subcutaneous tumors (NIR fluorescence imaging). (B) Immunofluorescence analysis of tumor tissue for BRD4, Bcl-2 and PD-L1 proteins.

To maximize the anti-tumor effect, increasing drug concentration at the tumor site is critical. Accordingly, we employed an NIR imaging system to evaluate the *in vivo* tumor-targeting capability of liposomes using the hydrophobic dye DiR as a tracer. As illustrated in Fig. 5A, both DiR@lip and DiR@Gip displayed pronounced fluorescence signals at tumor sites. However, the fluorescence intensity in the DiR@Gip group was significantly higher than that in the DiR@lip group. *Ex vivo* imaging further confirmed these findings, revealing stronger tumor-specific fluorescence (Fig. 5A and Fig. S6) in DiR@Gip-treated mice compared to the DiR@lip group. These results suggested that DiR@Gip exhibited superior tumor-targeting efficiency over DiR@lip, which contrasts with previous reports [21]. This enhanced targeting may be attributed to: (1) passive accumulation *via* the EPR effect [43], (2) prolonged circulation time due to GRh2 modification [22,44], and (3) active targeting mediated by GLUT1 [21].

To validate these observations, we next assessed the intra tumoral distribution of C6-loaded liposomes. Consistent with the NIR imaging data, tumor sections from C6@Gip-treated mice exhibited stronger green fluorescence than those from the C6@lip group (Fig. S7), further confirming the improved targeting ability of Gip. These findings imply that Gip can deliver higher payloads of ARV825 to tumor tissues compared to traditional liposomes.

Subsequently, we investigated whether ARV@Gip could effectively degrade BRD4 protein at the tumor site. Immunofluorescence analysis (Fig. 5B) demonstrated significantly greater BRD4 degradation in the ARV@Gip group than in the ARV@lip group. Consequently, the expression levels of downstream proteins (Bcl-2 and PD-L1) were also markedly reduced in ARV@Gip-treated tumors. The Western blot analysis of BRD4, Bcl-2, and PD-L1 protein expression in tumor tissues (Fig. S8) yielded results consistent with those obtained by immunofluorescence.

In summary, Gip outperformed lip in tumor-targeting efficiency, enabling enhanced delivery of ARV825 to tumor tissues. This leaded to effective BRD4 degradation and subsequent downregulation of Bcl-2 and PD-L1, highlighting the therapeutic potential of GRh2-functionalized nanocarriers.

### 3.6 Enhanced antitumor efficacy of ARV@Gip in subcutaneous tumor models

**Fig. 6:**
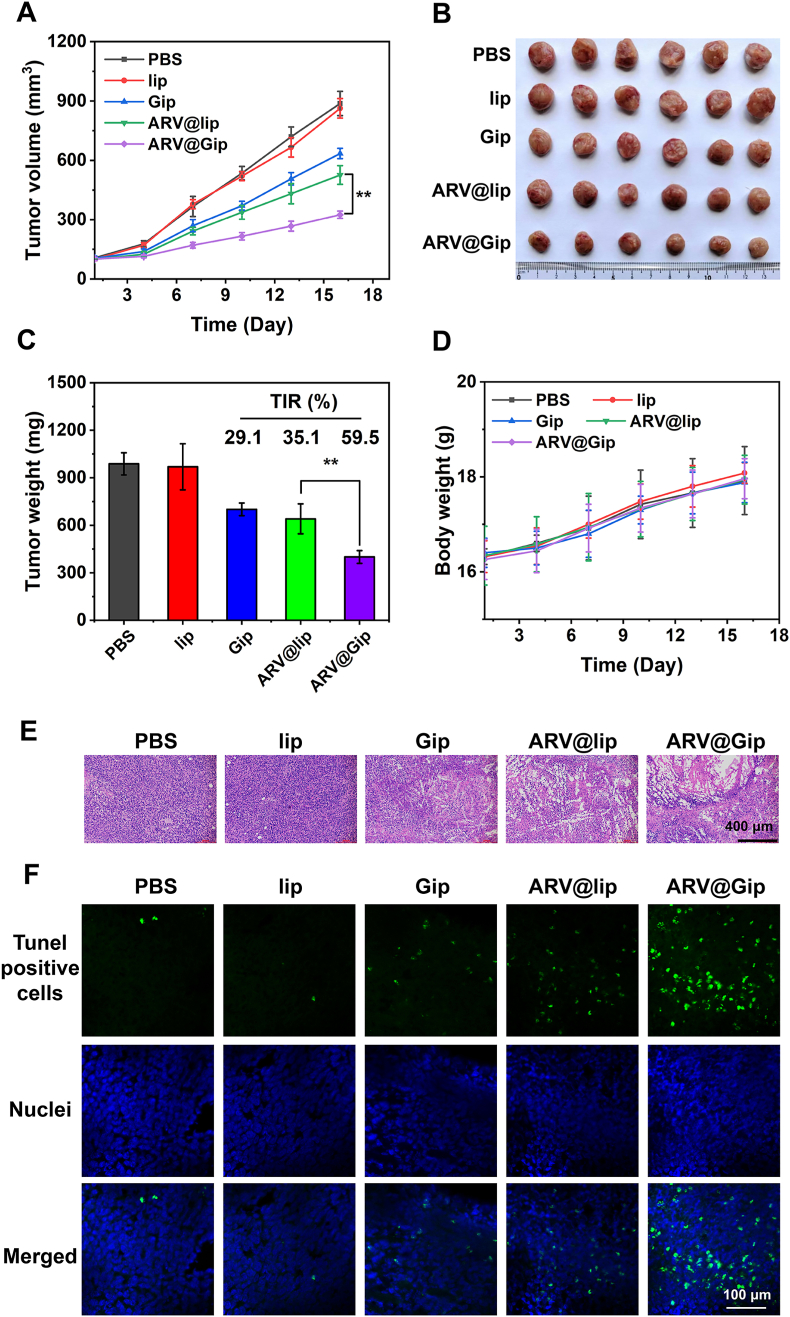
Therapeutic evaluation in subcutaneous 4T1 tumor models. Mice were injected *via* the tail vein on days 1, 4, 7, and 10: (1) PBS, (2) Lip, (3) Gip, (4) ARV@lip, or (5) ARV@Gip (Dose of ARV825 was 8 mg/kg). (A) Longitudinal tumor growth curves (n = 6/group). (B) Excised tumor specimens at endpoint. (C) Final tumor weights. (D) Treatment-associated body weight changes. (E) Histopathological assessment (H&E staining) of tumor tissues. (F) Apoptosis detection of tumor tissues *via* TUNEL assay.

The antitumor activity and systemic toxicity of ARV@Gip were evaluated through continuous monitoring of subcutaneous tumor volumes and body weight changes. As illustrated in Fig. 6A–C and Fig. S9 (individual tumor growth curves), blank lip showed minimal impact on tumor growth, while Gip demonstrated moderate tumor growth inhibition. Comparative analysis revealed that ARV@Gip exhibited significantly superior antitumor effects compared to ARV@lip, likely attributable to enhanced tumor targeting capability and synergistic interaction between GRh2 and ARV825. Consequently, ARV@Gip achieved significantly higher tumor inhibition rates than other treatment groups.

Histopathological examination of tumor tissues showed the most extensive necrotic areas in the ARV@Gip group through H&E staining, while TUNEL assays revealed the highest frequency of apoptotic cells in this treatment group (Fig. 6E and F). Importantly, neither ARV@Gip nor ARV@lip administration resulted in significant body weight loss (Fig. 6D). Furthermore, all measured serum biochemical indices (e.g., ALT, AST, BUN, CREA) in the treatment groups remained within normal physiological ranges and showed no statistically significant differences compared to the PBS control group (Fig. S10). Consistent with these findings, H&E staining of major organs revealed no evidence of necrosis or other notable pathological changes across all groups (Fig. S11). Together, these results indicate that GRh2-functionalized liposomes can enhance therapeutic efficacy while maintaining a favorable safety profile [45].

**Fig. 7:**
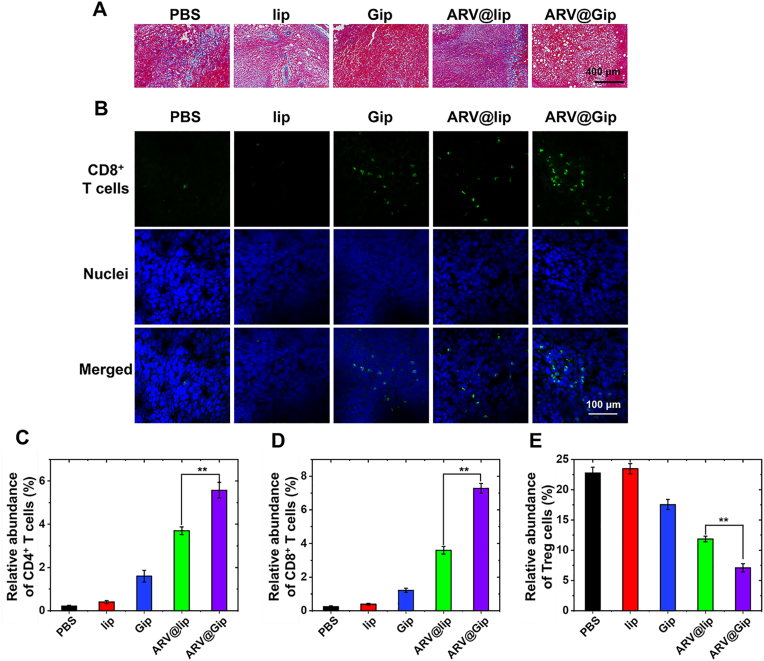
Tumor microenvironment modulation. (A)Masson's trichrome staining (collagen deposition) and (B)immunohistochemical analysis of CD8^+^ T cell infiltration. (C–E) Distribution of CD4^+^ T cells, CD8^+^ T cells, and Tregs in tumor tissues post-treatment, studied by flow cytometry.

Further characterization of tumor ECM modulation (Fig. 7A) demonstrated that Gip treatment substantially reduced stromal collagen deposition compared to both PBS and lip controls. ARV@Gip also showed more pronounced collagen attenuation relative to ARV@lip, which had no detectable effect on collagen remodeling despite its antitumor activity. Immunological analysis revealed markedly increased CD8^+^ T cell infiltration in Gip-treated tumors compared to lip controls, with ARV@Gip showing the most robust T cell penetration (Fig. 7B) [21,46]. To further substantiate the immunomodulatory effects, we have performed additional flow cytometry analysis of tumor tissues (Fig. 7C–E and Fig. S12). The results confirm that ARV@Gip treatment led to a significant increase in the infiltration of CD4^+^ and CD8^+^ T cells and a concurrent decrease in regulatory T cells (Tregs).

This enhanced anti-tumor immune profile supports our conclusion that the improved efficacy is not solely due to ECM remodeling, but arises from a multifaceted mechanism: (1) the intrinsic immunomodulatory effects of GRh2, and (2) the significant downregulation of PD-L1 on tumor cells by the payload ARV825, which collectively reprogram the immunosuppressive tumor microenvironment [25,47].

### 3.7 Targeted therapeutic efficacy of ARV@Gip against lung metastases of TNBC

**Fig. 8:**
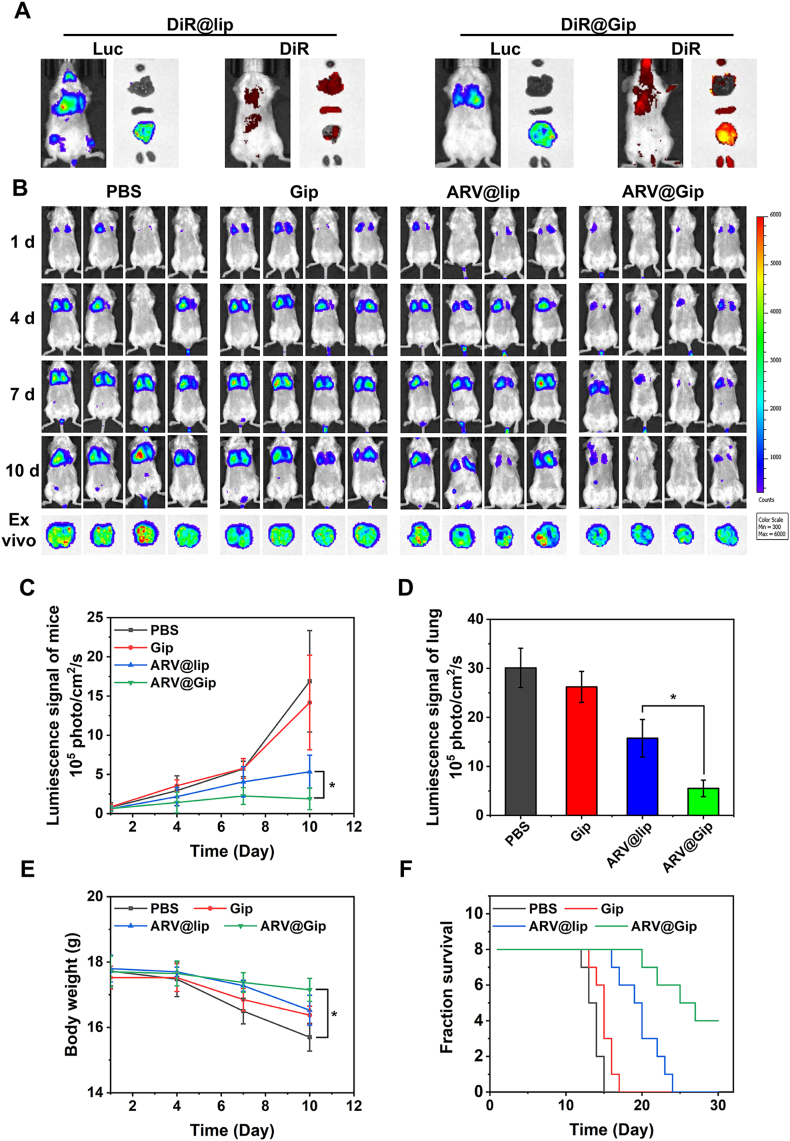
Targeting therapeutic efficacy of ARV@Gip in TNBC lung metastasis. (A) NIR imaging of DiR-liposome accumulation in metastatic lungs. (B) Longitudinal BLI of metastatic progression. (C) Quantitative BLI signal kinetics. (D) *Ex vivo* lung BLI quantification. (E) Treatment-associated body weight dynamics. (F) Kaplan-Meier survival analysis.

The pronounced metastatic propensity of TNBC, particularly to lung tissues, represents a major therapeutic challenge [48]. To investigate the tumor-targeting potential and antimetastatic efficacy of ARV@Gip, we developed an experimental lung metastasis model through intravenous administration of 4T1-Luc cells. NIR fluorescence imaging demonstrated superior accumulation of DiR@Gip in lung metastatic lesions compared to DiR@lip, with significantly enhanced fluorescence intensity in the Gip-treated group (Fig. 8A and Fig. S13), indicating remarkable targeting specificity toward TNBC lung metastases. Previous investigations have established the active targeting capability of ginsenoside Rg3 (GRg3)-modified liposomes against TNBC lung metastases [35]. Considering the structural similarity between GRh2 and GRg3, especially their shared glycosylation patterns, we proposed that the glycosylated configuration of GRh2 in Gip conferred comparable active targeting properties. This molecular architecture likely facilitates selective accumulation in metastatic TNBC lesions within lung tissue while simultaneously exerting antitumor effects, thereby offering a dual therapeutic mechanism against metastatic progression.

Subsequent evaluation of ARV@Gip's efficacy against TNBC lung metastases yielded compelling results. As shown in Fig. 8B–D, bioluminescence imaging revealed rapid metastatic progression in untreated controls, accompanied by severe body weight loss and increased mortality (Fig. 8E and F) [35]. While both ARV@lip and ARV@Gip attenuated metastasis, ARV@Gip exhibited superior suppression of lung lesions, consistent with its enhanced performance in subcutaneous tumors. H&E staining (Fig. S14) further corroborated ARV@Gip's efficacy, showing significantly reduced lung tumor burden versus other groups [49]. Critically, ARV@Gip treatment mitigated cancer-associated wasting and markedly prolonged survival. Together, these findings establish ARV@Gip as a dual-action therapeutic, effectively controlling both subcutaneous tumors and lung metastases of TNBC.

## 4 Conclusion

In this study, we developed GRh2-functionalized liposomes for efficient targeted delivery of BRD4-PROTAC *in vivo*, demonstrating therapeutic efficacy against both subcutaneous breast cancer tumors and lung metastases. This innovative combination potentiated the antitumor activity of ARV825 through multiple mechanisms: (1) Enhanced tumor accumulation and cellular internalization of ARV825 *via* GLUT1-mediated active targeting; (2) Induction of tumor cell apoptosis through downregulation of Bcl-2 protein mediated by BRD4 protein degradation; (3) Promotion of T cell infiltration and antitumor immune response *via* BRD4 degradation-mediated PD-L1 suppression combined with GRh2-mediated collagen fiber degradation in the tumor ECM. Furthermore, this drug delivery system exhibited excellent biosafety profiles with no observed systemic toxicity. In conclusion, the GRh2-functionalized liposomal platform developed in this study provided a promising strategy for effective BRD4-PROTAC delivery in breast cancer treatment, addressing critical challenges in current targeted therapies through its multifunctional design.

## CRediT authorship contribution statement

**Lijuan Wen:** Project administration, Funding acquisition, Formal analysis, Data curation. **Jialei Rao:** Writing – review & editing, Validation, Supervision. **Jiaoting Chen:** Resources, Methodology, Investigation. **Fang Li:** Visualization, Methodology. **Xixi Chen:** Visualization, Validation, Formal analysis. **Shenpeng Guo:** Formal analysis, Data curation. **Binghui Cui:** Validation, Investigation, Data curation. **Caisheng Qiu:** Writing – review & editing, Validation, Resources. **Weiliang Chen:** Writing – original draft, Project administration, Funding acquisition.

## Declaration of competing interest

The authors declare that they have no known competing financial interests or personal relationships that could have appeared to influence the work reported in this paper.

## Data Availability

Data will be made available on request.

## Abbreviation list

PROTAC: Proteolysis-Targeting Chimera
ARV825: A BRD4-targeting PROTAC molecule
BRD4: Bromodomain-containing protein 4
Bcl-2: B-cell lymphoma 2
PD-L1: Programmed Death-Ligand 1
ECM: Extracellular matrix
lip: Conventional liposomes
GRh2: Ginsenoside Rh2
Gip: Ginsenoside Rh2-functionalized liposomes
ARV@Gip: ARV825-loaded Ginsenoside Rh2-functionalized liposomes
ARV@lip: ARV825-loaded conventional liposomes
GLUT1: Glucose transporter 1
UPS: Ubiquitin-Proteasome System
BET: Bromodomain and Extra-Terminal motif
EPR: Enhanced Permeability and Retention effect
SPC: Soy phosphatidylcholine
DiR: 1,1′-Dioctadecyl-3,3,3′,3′-Tetramethylindotricarbocyanine Iodide
MTT: 3-(4,5-Dimethylthiazol-2-yl)-2,5-diphenyltetrazolium bromide
FITC: Fluorescein isothiocyanate
TUNEL: Terminal deoxynucleotidyl transferase dUTP nick end labeling
4T1-Luc: Luciferase-expressing 4T1 cells
DLS: Dynamic Light Scattering
PDI: Polydispersity Index
EE%: Encapsulation Efficiency
DL%: Drug Loading capacity
HPLC: High-Performance Liquid Chromatography
C6: Coumarin-6
C6@Gip: Coumarin-6-labeled Ginsenoside Rh2-functionalized liposomes
C6@lip: Coumarin-6-labeled conventional liposomes
CLSM: Confocal Laser Scanning Microscopy
PBS: Phosphate-Buffered Saline
DAPI: 4′,6-Diamidino-2-phenylindole
PI: Propidium Iodide
CCK-8: Cell Counting Kit-8
NIR: Near-Infrared
DiR@Gip: DiR-labeled Ginsenoside Rh2-functionalized liposomes
DiR@lip: DiR-labeled conventional liposomes
AST: Aspartate Aminotransferase
ALT: Alanine Aminotransferase
BUN: Blood Urea Nitrogen
CREA: Creatinine
H&E: Hematoxylin and Eosin
TNBC: Triple-Negative Breast Cancer
BLI: Bioluminescence Imaging
IC50: Half Maximal Inhibitory Concentration
Tregs: Regulatory T cells
L929: Mouse fibroblast cell line
DMSO: Dimethyl sulfoxide
FBS: Fetal Bovine Serum
SD: Standard Deviation
ANOVA: Analysis of Variance
