# Supplementary material for: Ginsenoside Rh2- functionalized liposomes enhanced BRD4-PROTAC delivery and antitumor efficacy via improved tumor targeting and ECM remodeling
Source: Mater Today Bio. 2026 Jan 5;36:102767. doi: 10.1016/j.mtbio.2026.102767 (PMC12818118; doi:10.1016/j.mtbio.2026.102767)
Supplement: Multimedia component 1 [file mmc1.docx]

**Supplementary Material**

**Ginsenoside Rh2-** **Functionalized Liposomes Enhanced BRD4-PROTAC Delivery and Antitumor Efficacy *via* Improved Tumor Targeting and ECM Remodeling**

Lijuan Wen^1,2#^, Jialei Rao^2#^, Jiaoting Chen^2#^, Fang Li^3^, Xixi Chen^2^, Shenpeng Guo^2^, Binghui Cui^2^, Caisheng Qiu^2^, Weiliang Chen^1,2*^

^1^Key Laboratory of Prevention and Treatment of Cardiovascular and Cerebrovascular Diseases of Ministry of Education, Gannan Medical University, University Park in Rongjiang New District, Ganzhou 341000, People’s Republic of China

^2^ College of Pharmacy, Gannan Medical University, University Park in Rongjiang New District, Ganzhou 341000, People’s Republic of China

^3^ Department of Pharmacy, Children’s Hospital of Soochow University, Suzhou, 215003, China

# These three authors contributed equally to this paper.

* Corresponding Authors

Weiliang Chen- College of Pharmacy, Gannan Medical University, University Park in Rongjiang New District, Ganzhou 341000, People’s Republic of China (E-mail: [chenweiliang2008@126.com](mailto:chenweiliang2008@126.com))


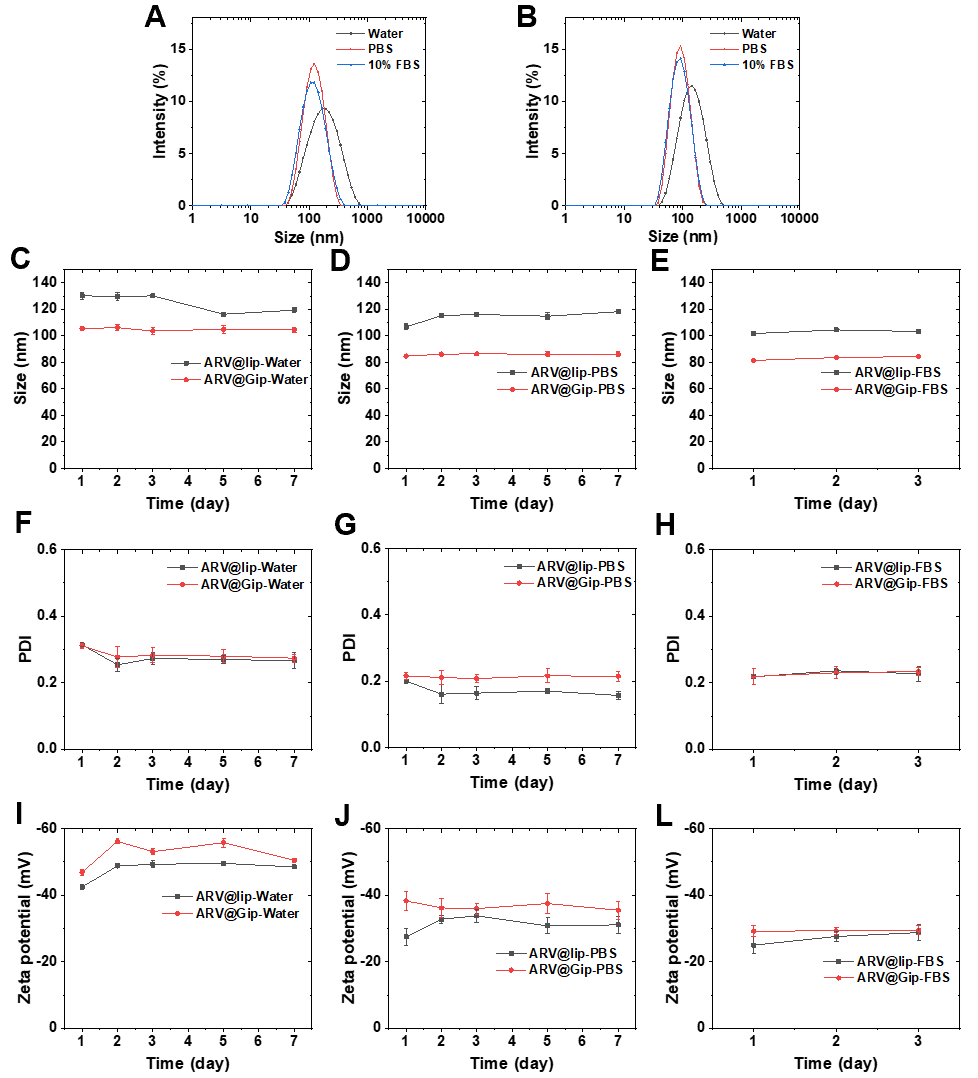
**Fig.S1** Stability of ARV@lip and ARV@Gip. A and B Particle size distribution of ARV@lip and ARV@Gip in water, PBS and 10% FBS. C-E Particle size changes of ARV@lip and ARV@Gip in water, PBS and 10% FBS. F-H PDI changes of ARV@lip and ARV@Gip in water, PBS and 10% FBS. I-L.Zeta potential changes of ARV@lip and ARV@Gip in water, PBS and 10% FBS.


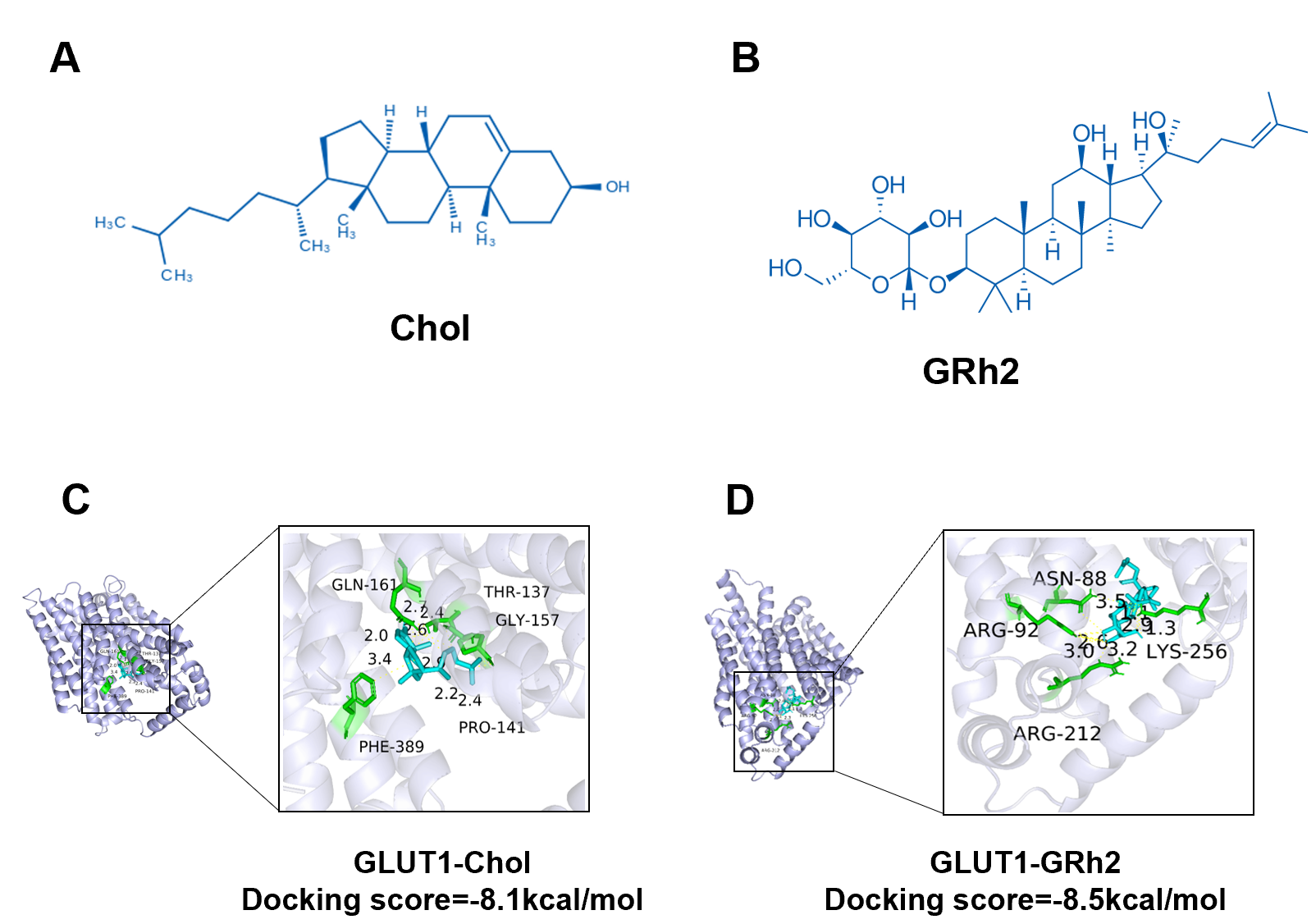


**Fig.S2** Chemical structure and molecular docking studies of GLUT1-Chol and GLUT1-GRh2


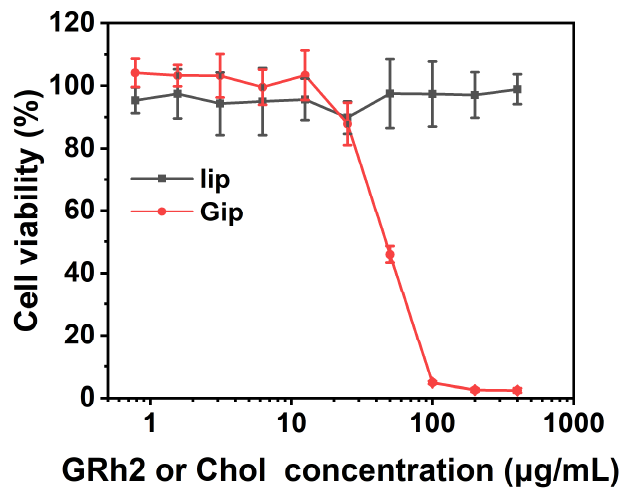


**Fig.S3** Cytotoxicity of blank lip and Gip studied by MTT assay.


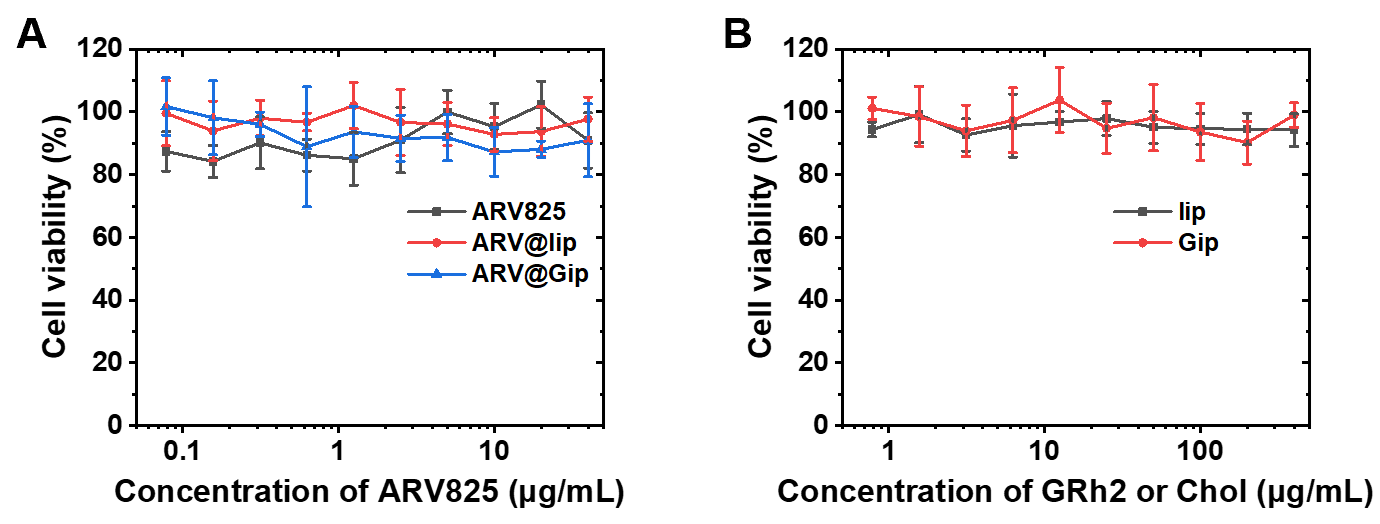


**Fig.S4** Cytotoxicity ARV825, ARV@lip, ARV@Gip, lip and Gip on hepatocytes (AML12 cells) (Studied using MTT method).


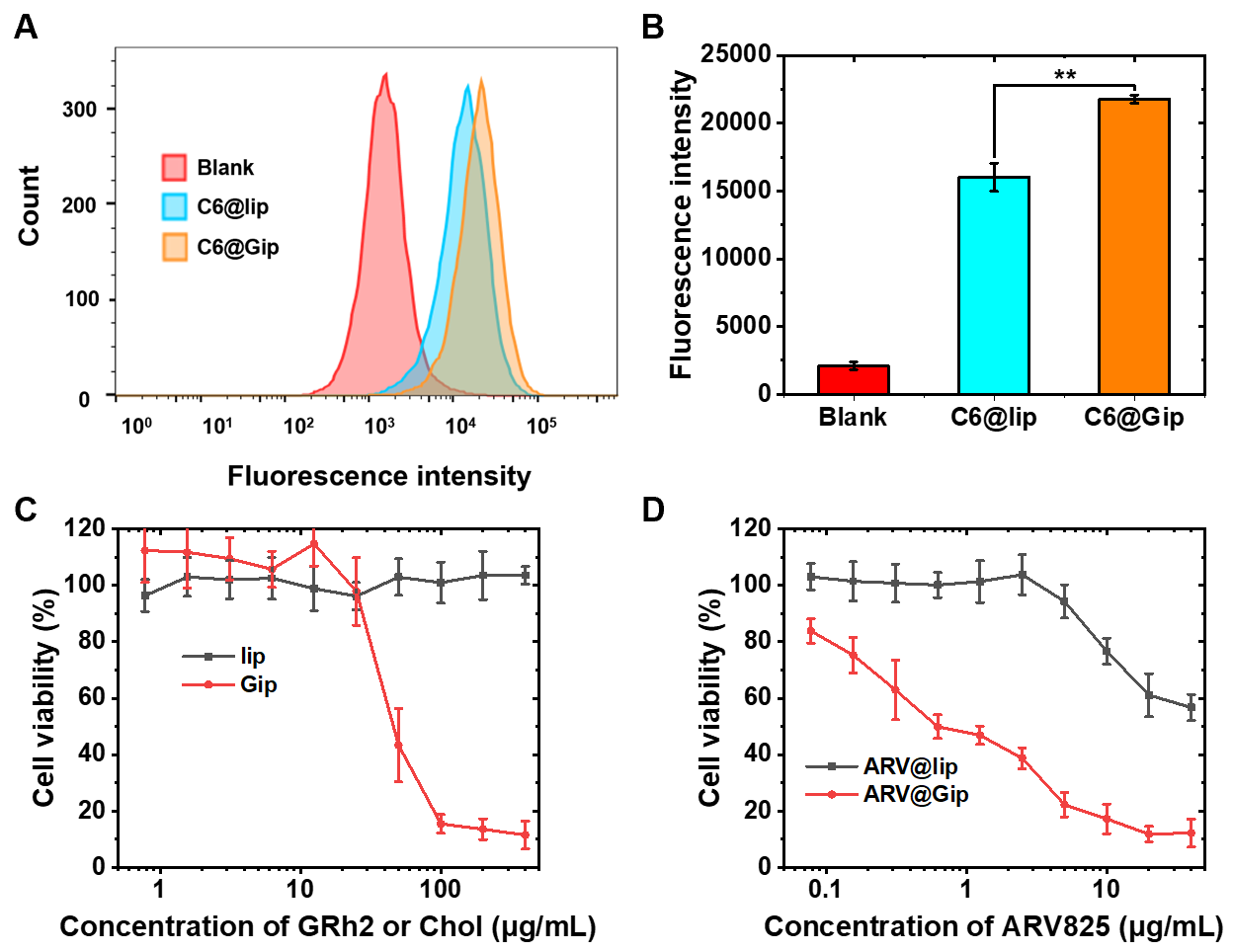


**Fig.S5** Cellular uptake and cytotoxicity to fibroblasts (L929 cells)


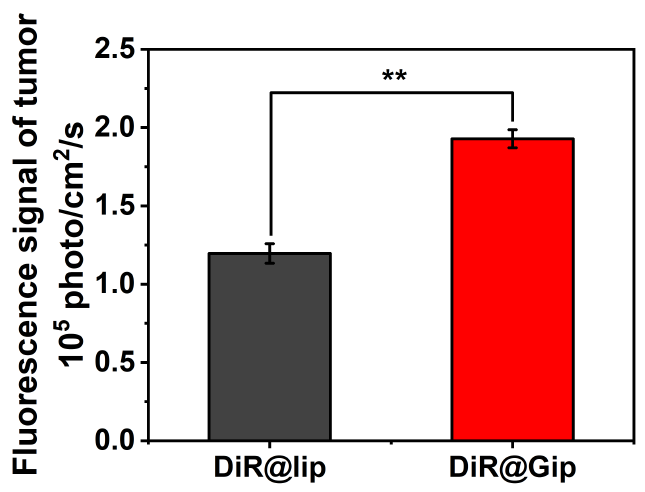


**Fig.S6** Quantitative analysis of DiR signals in isolated tumor tissues


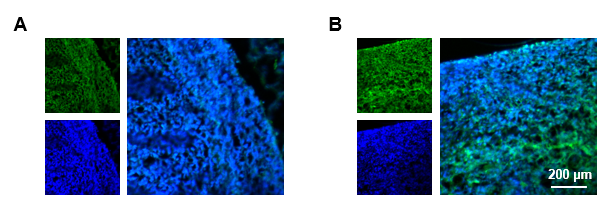


**Fig.S7** Distribution of C6@lip (A) and C6@Gip (B) in tumor tissues


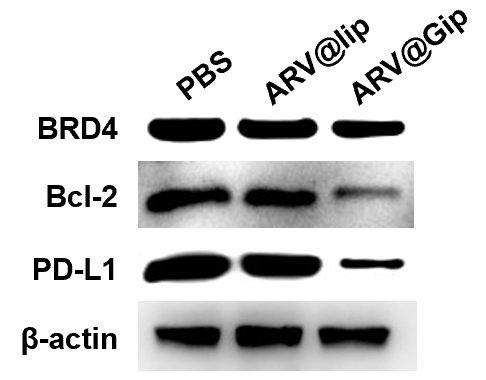


**Fig.S8** Study of BRD4, Bcl-2, and PD-L1 in tumor tissues using WB.


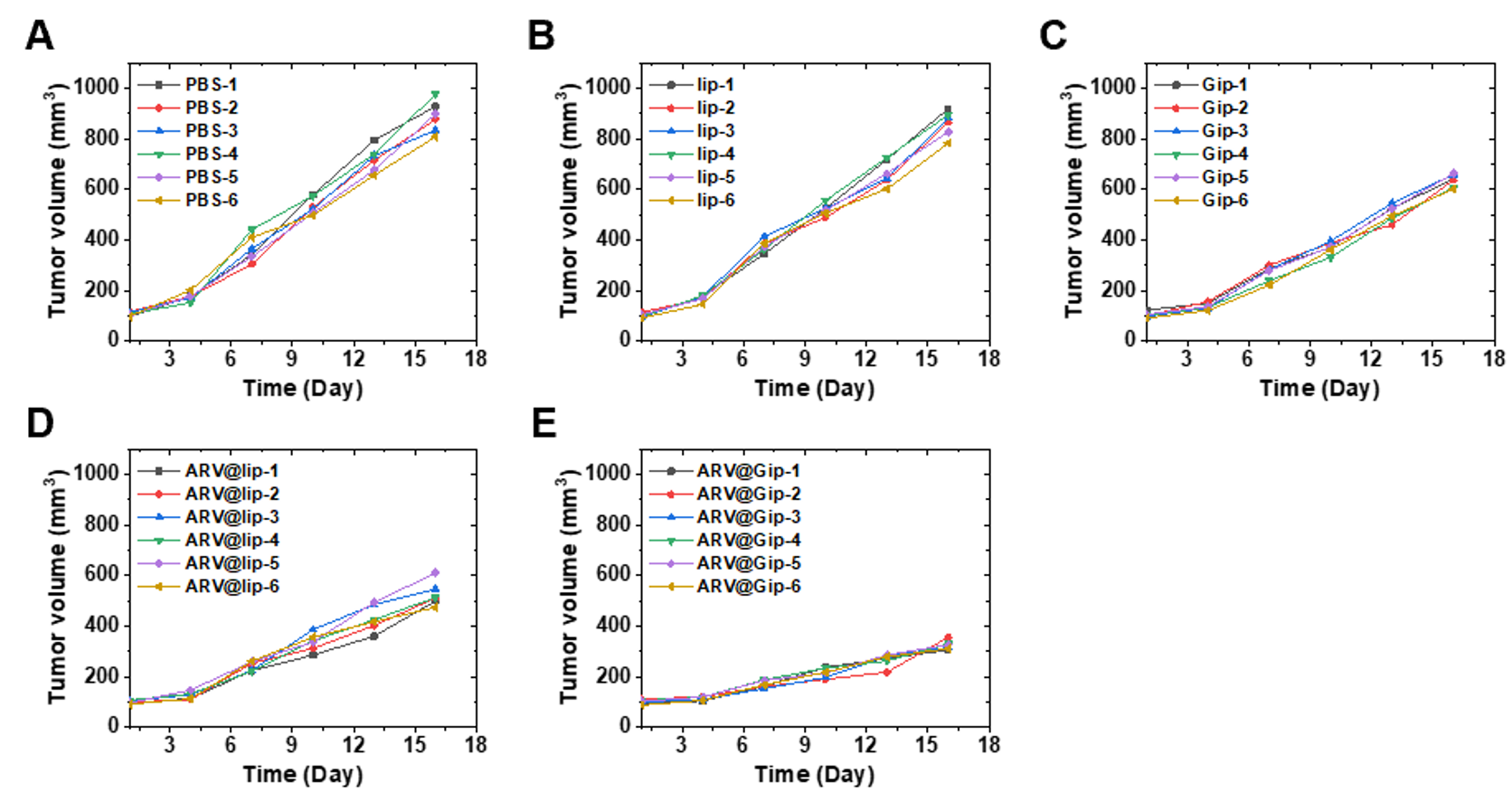


**Fig.S9** individual tumor growth curves


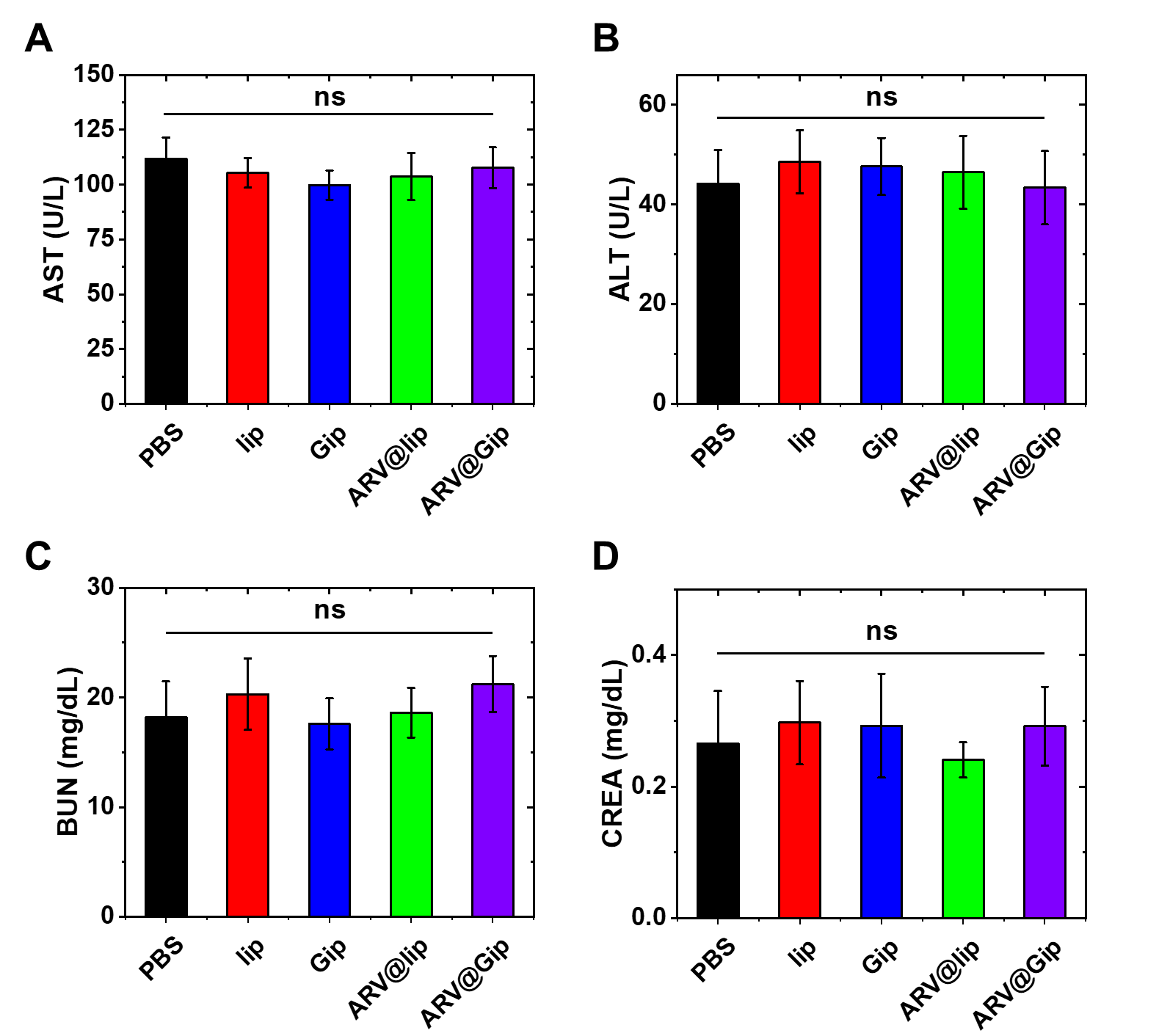


**Fig. S10** Serum biochemical parameters (ALT, AST, BUN, creatinine) after the treatment


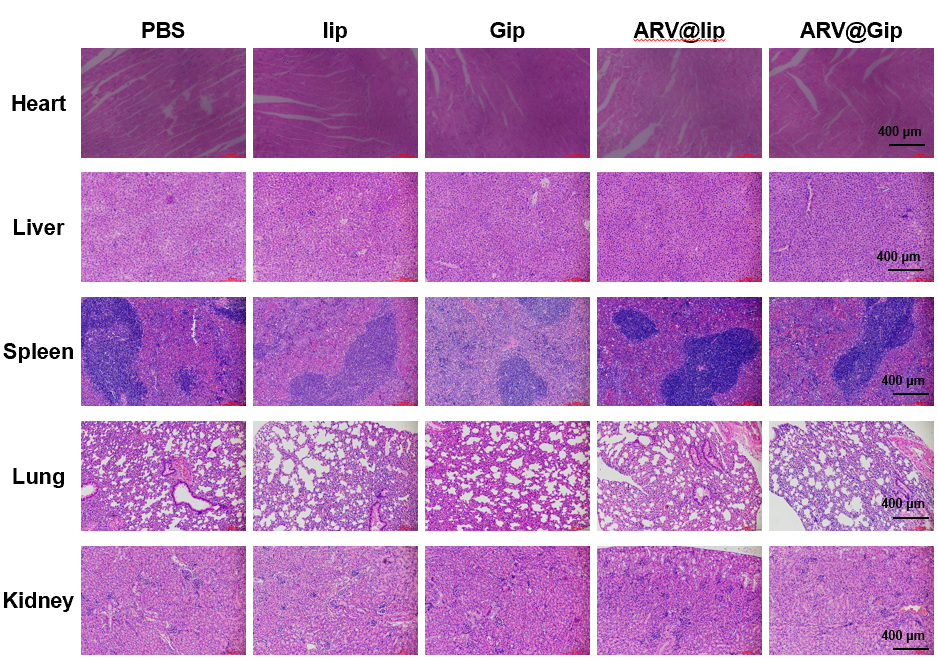
**Fig.S11** H&E staining of main organs after the treatment.


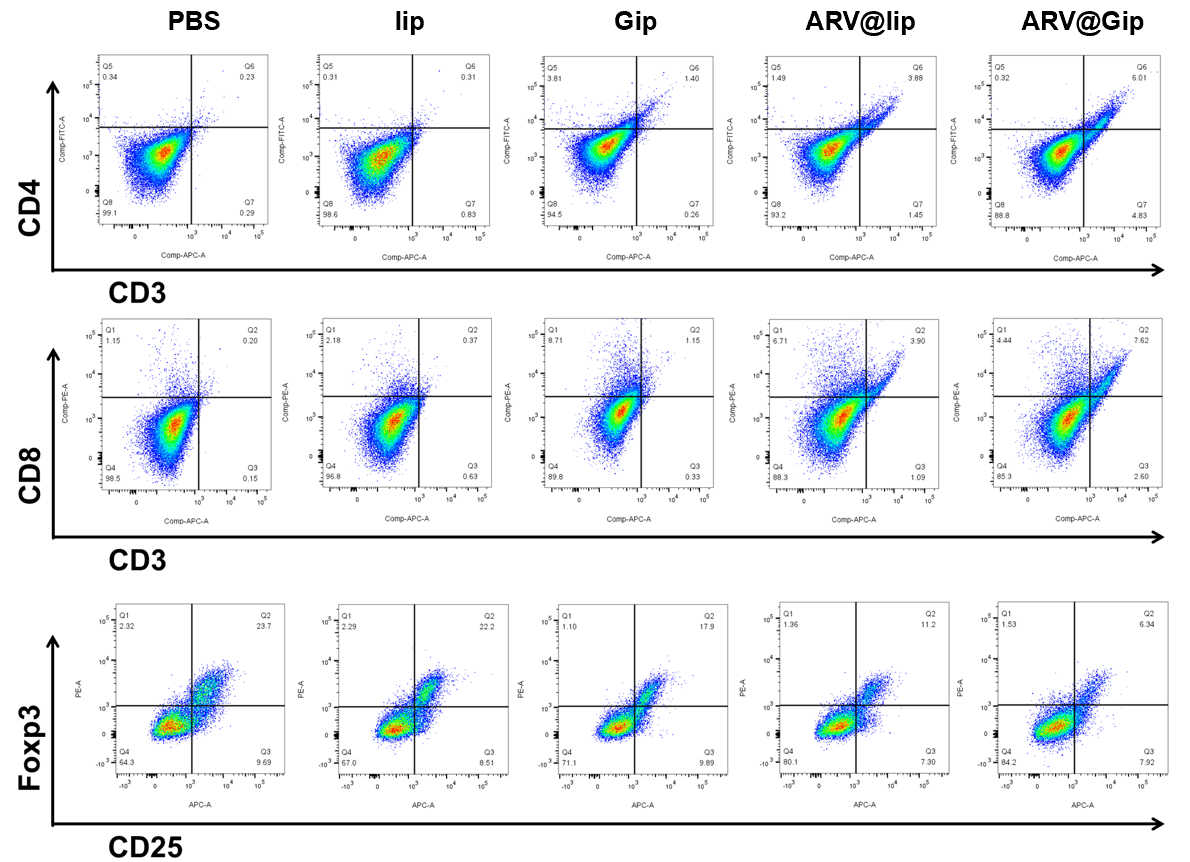
**Fig.S12** Distribution of CD4^+^ T cells, CD8^+^ T cells, and Tregs in tumor tissues post-treatment.


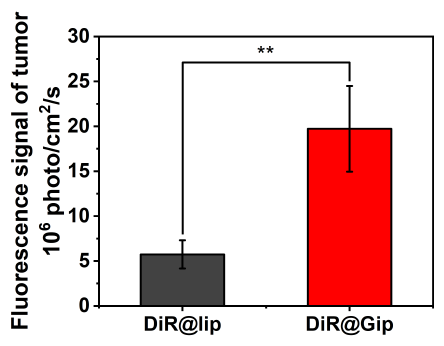


**Fig.S13** Quantitative analysis of DiR signals in isolated lung tissues


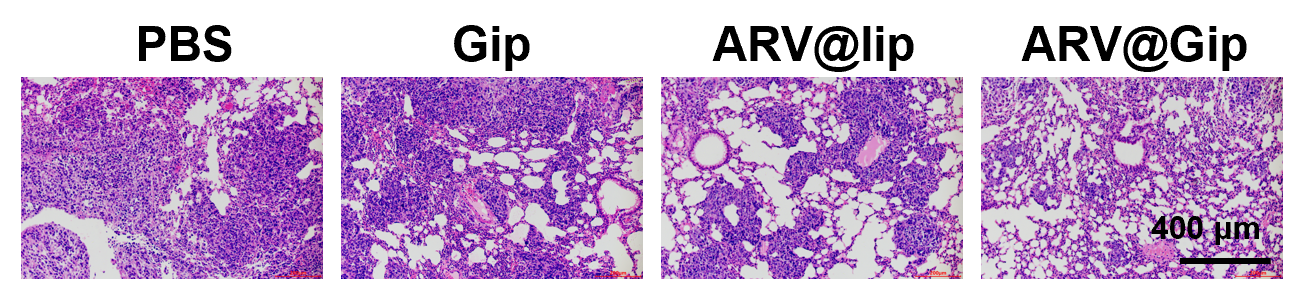


**Fig.S14** H&E staining of lung after the treatment.
